# Accuracy of Soluble Endoglin for Diagnosis of Preeclampsia and its Severity

**DOI:** 10.18869/acadpub.ibj.21.5.312

**Published:** 2017-09

**Authors:** Pooneh Nikuei, Minoo Rajaei, Kianoosh Malekzadeh, Azim Nejatizadeh, Fatemeh Mohseni, Ali AtashAbParvar

**Affiliations:** 1 Molecular Medicine Research Center, Hormozgan Health Institute, Hormozgan University of Medical Sciences, Bandar Abbas, Iran; 2 Fertility and Infertility Research Center, Hormozgan University of Medical Sciences, Bandar Abbas, Iran; 3 Department of Medical Genetics; Faculty of Medicine; Hormozgan University of Medical Sciences, Bandar Abbas, Iran; 4 Pathology Department, Faculty of Medicine, Hormozgan University of Medical Sciences, Bandar Abbas, Iran

**Keywords:** Preeclampsia, Endoglin, Biomarkers, Pregnancy

## Abstract

**Background:**

The use of biomarkers for diagnosis of Preeclampsia (PE), a life-threatening pregnancy disorder, could reduce serious complications of this disease. In this study, we investigated dysregulation of endoglin (Eng) expression and diagnostic accuracy of soluble endoglin (sEng) in PE patients.

**Methods:**

For this case-control study, 26 mild and 15 severe preeclamptic women along with 20 normotensive controls were recruited. The expression level of Eng (the co-receptor of TGF-β1) was evaluated using qRT-PCR. Also, the serum concentration of soluble Eng and expression of membranous Eng were determined by ELISA and immunohistochemistry.

**Results:**

A significant up-regulation in Eng mRNA and sEng levels was observed in PE patients versus normal controls. Immunohistochemistry (IHC) showed up-regulation of membranous Eng staining in syncytiotrophoblast and cytotrophoblast cells of PE patients. The serum levels of sEng were significantly increased in all patients (mild, sever, early- and late-onset) as compared to healthy pregnant women (*P*<0.001). Receiver-operating characteristic (ROC) curve analysis revealed that sEng had the highest accuracy in distinguishing PE from normal pregnancies with cut-off value of 20.4, sensitivity of 92.1%, specificity of 90%, and area under the curve (AUC) of 0.94 (95% CI: 0.88-1.00).

**Conclusions:**

Our data showed that the up-regulation of Eng mRNA along with its membranous and soluble form in PE patients leads to defect in angiogenesis pathway. Also, the results of this study revealed sEng potential as a marker for diagnosis of PE and its severity.

## INTRODUCTION

Preeclampsia (PE) is one of the most critical complications of pregnancy and characterized by hypertension and proteinuria after 20 weeks of gestation[1]. It affects 5-8% of all pregnancies and is responsible for 60,000 maternal deaths and more fetal and neonatal deaths every year[2]. PE imposes a considerable expenses on patients and healthcare system[3]. In order to reduce serious complications of PE and early treatment interventions, initial diagnosis of high risk women for developing PE is important. Biomarkers for prediction of PE before manifestation of clinical symptoms in early pregnancy would help to identify such cases for more careful follow-up and early interventions[4]. Currently, there is no definite treatment for PE, except preterm delivery of placenta and fetus, which increases the risk of disability and death of the newborn, especially in severe early-onset cases[5].

Despite uncertain etiology of PE, angiogenesis defect in the early stages of pregnancy results in incomplete remodeling of uterine spiral arterioles, abnormal placental vascular development, and endothelial dysfunction as the main causes of PE[6-8]. Various studies have indicated the role of soluble endoglin (sEng), an anti-angiogenic factor, in the etiology of PE[1,9].

Eng or CD105 is a homodimeric transmembrane glycoprotein that is localized on cell surfaces and acts as a co-receptor for transforming growth factor (TGF)-β1 and TGF-β3 isoforms[10]. Eng is known to have a main function in vascular tone maintenance via the regulation of nitric oxide dependent vasodilatation, and probably control placental implantation and spiral artery remodeling during pregnancy[11]. Involvement of Eng in PE has already been studied based on its role in systemic endothelial dysfunction, shallow placental implantation, and spiral artery remodeling demonstrated as principal pathophysiologic abnormalities in this disease[11]. This study was designed to evaluate the expression alterations of Eng as well as to assess the usefulness of sEng as a diagnostic biomarker for PE patients.

## MATERIALS AND METHODS

### Patients and sampling

A total of 61 pregnant women, 41 preeclamptic cases and 20 normotensive controls, were recruited for this case-control study. All participants were selected from two hospitals in Bandar Abbas and were provided written informed consent for serum and placental tissue collection under the protocols approved by the Ethical Committee of Hormozgan University of Medical Sciences (No. 1-HEC-93-7-8) from 2014 to 2015. Participants did not receive any medication before sampling. Preeclamptic women were divided into mild (n=26) and severe (n=15), and cases were subdivided in 9 early- and 32 late-onset PE, respectively.

PE definition was determined based on the American Congress of Obstetricians and Gynecologists guidelines as: gestational hypertension (systolic pressure >140 mmHg or diastolic blood pressure >90 mmHg on two or more occasions after gestational week 20) with proteinuria (>0.3 g/day). Severe PE was defined if more than one of the following criteria were present: (i) severe gestational hypertension (systolic pressure>160mm Hg or diastolic blood pressure>110 mm Hg on two or more occasions after gestational week 20), (ii) severe proteinuria (≥5 g of protein in a 24-h urine specimen), (iii) oliguria <500 ml in 24 h, (iv) cerebral or visual disturbances, (v) pulmonary edema or cyanosis, (vi) epigastric or right upper-quadrant pain, (vii) impaired liver function, (viii) thrombocytopenia or (ix) fetal growth restriction[12]. Early-onset PE was considered as (<34+0 weeks) and late-onset PE as (≥34+0 weeks)[13].

Women with diabetes, collagen vascular diseases, renal disease, chronic or gestational hypertension, and fetal anomalies were excluded from the study. Immediately after delivery and after removal of chorionic plate and overlaying fetal membranes, small pieces of tissues biopsies (2 cm^3^ each) were randomly obtained from about 2 cm beside the umbilical cord insertion and from areas without infarction and hemorrhage. Placental tissues were washed in sterile PBS and placed in tubes containing RNAlater solution (Qiagen, Germany) and stored at -80°C until RNA extraction. Placenta samples of 15 women (5 mild, 5 severe, and 5 normal) were immersion-fixed in 10% formalin and after that embedded in paraffin wax. The whole blood samples were centrifuged at 3000 ×g for 20 min, and the separated serum samples were stored at -80°C for ELISA.

### RNA extraction

Total RNA was extracted from placental samples (approximately 100 mg) using TRIZOL reagent (Sigma-Aldrich, USA). The extracted RNA was then treated with RNase-free DNase-I (Thermo Scientific, USA) according to the manufacturer’s instructions. The quality and quantity of the extracted RNA were evaluated by agarose gel electrophoresis and spectrophotometery (NanoDrop ND-1000, Thermo Scientific, USA), respectively.

### cDNA synthesize and quantitative real-time PCR (qRT-PCR)

Total RNA (2 µg) was reverse transcribed to cDNA by the RevertAid™ First Strand cDNA Synthesis Kit (Fermentas, Canada) using random hexamer primer following the manufacturer’s protocol. qRT-PCR was conducted by a real-time PCR system (Corbett, Rotor-Gene 6000, Australia) using specific primer sets for each gene (Table 1) and Syber Green-Master Mix (Takara Syber Premix Ex Taq, Japan) according to the manufacturer’s instructions. All reactions were set as 20-µl mixture containing 2 µl cDNA, 10 µl Master Mix 2×, 0.4 µl ROX dye 50× and 10 pmol of each primer pair for Eng, TGF-β1, and β-actin (1.6 µl). The thermal cycling status was initial denaturation at 95°C for 30 s, followed by 40 cycles of denaturation at 94°C for 5 s, annealing at temperatures for each primer pair for 15 s, extension at 60°C for 30 s. The expression levels of TGF-β1 and Eng were normalized by β-actin expression as house-keeping gene and calculated by the 2^-∆∆CT^ method.

**Table 1:** Primer sequences, amplicon sizes, and the annealing temperature in quantitative real-time PCR

Gene	Sequence of primers 5’→3’	Size (bp)	An. Temp. (°C)
*TGF-β1*	F: CGACTACTACGCCAAGGAGGT’	149	62
	R: AGAGCAACACGGGTTCAGGTA		
*Endoglin*	F: AGGCGGTGGTCAATATCC	109	62
	R: AAGTGTGGGCTGAGGTAG		
*β-actin*	F: GCCTTTGCCGATCCGC	90	58
	R: GCCGTAGCCGTTGTCG		

An.Temp., annealing temperature; F, forward primer; R, reverse primer

### ELISA

ELISA was performed on 58 serum samples including 23 mild PE patients, 15 severe PE patients, and 20 normal pregnancies. In case group, 9 women were affected with early-onset PE, and 29 women suffered from late-onset PE. Serum Eng level was determined by using a commercially available Quantikine Human Eng Immunoassay kit (R&D Systems, USA) according to the procedure provided by the manufacturer.

### Immunohistochemistry

IHC for Eng was performed on placental tissues obtained from preeclamptic and healthy pregnant women. Briefly, paraffin sections, which were mounted on glass slides, dewaxed in xylene and rehydrated using descending grade of ethanol (100, 96, and 70). Tissue sections were heated for 5 minutes at a 1000-W microwave then 15 minutes at 300-W and cooled at room temperature for 20 minutes. The sections were washed in PBS (pH 7.6) for 10 minutes and immersed in H_2_O_2_ 3% solved in methanol at room temperature for 10 minutes. The sections were then washed with PBS and incubated at 4-6°C overnight with 1:150 diluted primary rabbit polyclonal antibody Eng (H-300: sc-20632, Santa Cruz, USA). Afterwards, the sections were washed with PBS and ChemMate™ DAKO EnVision™ Detection Kit, peroxidase/3,3’-diaminobenzidine (DAB), rabbit/mouse (DakoCytomation, Denmark) was applied according to manufacturer’s instructions following staining with hematoxylin (DDK, Italia). For negative controls, primary antibody was deleted and for positive controls human kidney tissue was used. All slides were processed at the same time and were examined under a microscope (Olympus CX31RBSF, Japan).

### Statistical analysis

One-way ANOVA/Kruskal-Wallis test with Bonferroni correction was used for comparing the data between multiple groups and Mann-Whitney test/unpaired *t*-test for comparison between two groups based on Kolmogorov-Smirnov test for normality distribution. Data were shown as number (%) and mean (±SD). qRT-PCR data were analyzed by GraphPad prism software (version 5.0; GraphPad Software Inc., San Diego, CA). Dunnet’s post hoc test was used to compare the expression level between the groups. *P*<0.05 was considered statistically significant. The receiver-operating characteristic (ROC) curve analysis was carried out to show the cut-off serum levels of sEng in PE patients compared to normal pregnancies using statistical software STATA 11 (Texas, USA).

## RESULTS

There was no significant difference in maternal age (*P*=0.816) between case and control groups. Clinical characteristics are displayed in Table 2.

**Table 2:** Clinical characteristics of the women participated in the study

Parameter	PE (n=41)	Controls (n=20)	*P* value
Mental age (year)	27.20±5.66	26.85±4.84	0.816
Placental weight (g)	427±85.04	609±49.41	<0.001
BMI before pregnancy	23.82±3.88	23.31±3.49	0.385
SBP (mmHg)	15.61±1.58	11.38±0.67	0.001
DBP (mm Hg)	10.04±0.92	6.90±0.72	0.001
Parity			
Primiparous	19(46.3%)	9(45%)	0.921
Multiparous	22(53.7%)	11(55%)	
Previous PE	6(14.6%)	0	0.165

Results are presented as mean±SD. BMI, body mass index; SBP, systolic blood pressure; DBP, diastolic blood pressure; n, number; PE, preeclampsia

### Up-regulation of endoglin expression in preeclampsia patients

**Fig. 1:**
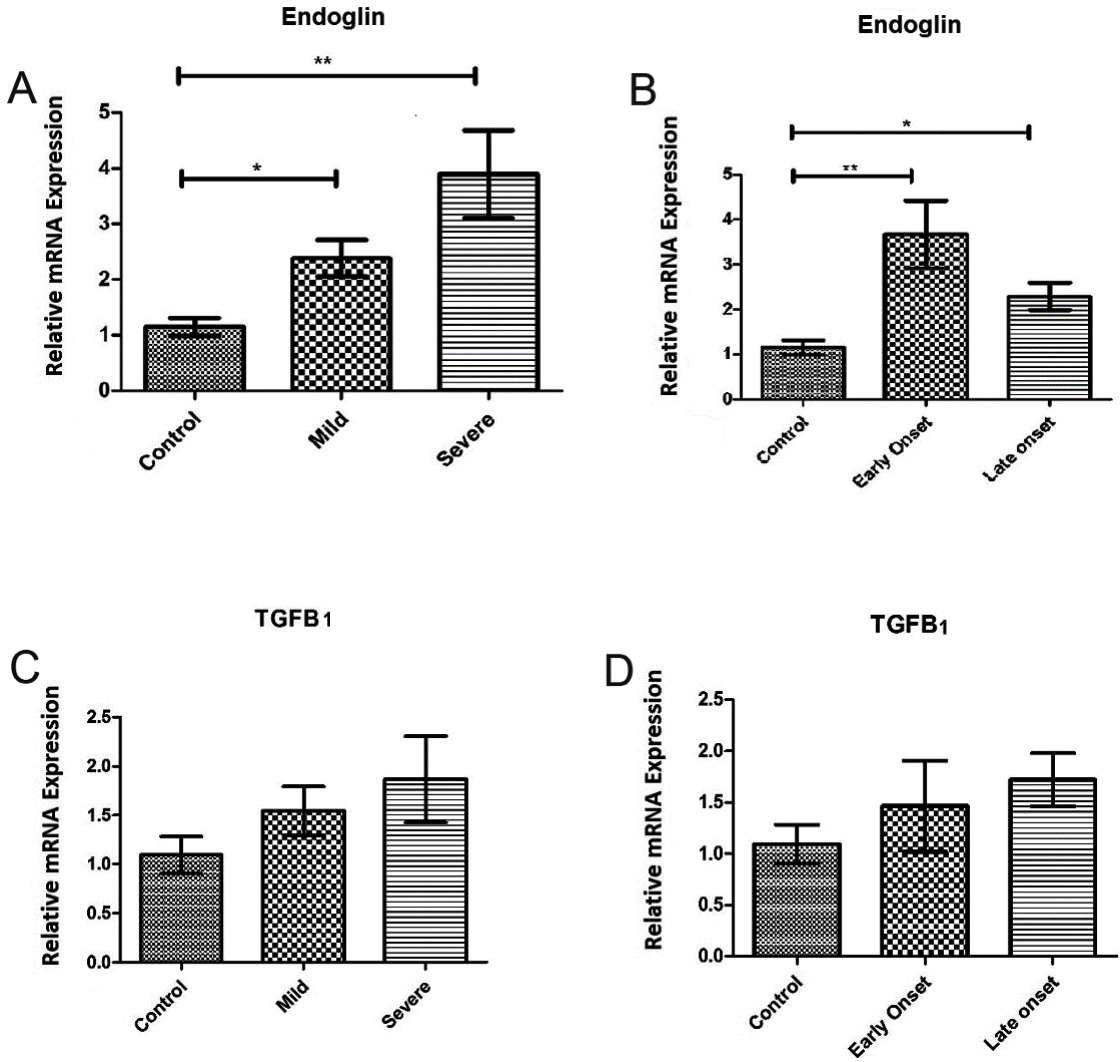
Placental expression of endoglin (Eng) and TGF-β1 mRNA in preeclampsia (PE) compared to uncomplicated term pregnancy. Eng mRNA expression among patients with mild and severe (A) as well as early- and late-onset PE (B) showed a significant up-regulation in all groups of patients in comparison with controls. TGF-β1 mRNA expression among patients with mild and severe (C) and also early- and late-onset PE (D) indicated no significant difference in comparison with controls. Values are presented as mean±S.E.M. ^*^P<0.05, ^**^P<0.01, respectively.

The result of mRNA expression showed no significant difference for TGF-β1 in PE patients compared with controls. Eng expression in mRNA level was significantly up-regulated in all groups of the patients compared to the controls, which was more significant in severe and early-onset PE patients (mild PE (*P*<0.05), severe PE (*P*<0.01), early-onset (*P*<0.01) late-onset (*P*<0.05). The altered expression of TGF-β1 and its co-receptor Eng in PE are displayed in Figure 1.

### Increased serum level of soluble endoglin in Preeclampsia patients

**Fig. 2:**
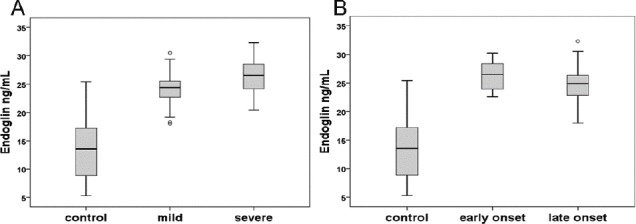
Levels of soluble Endoglin (sEng) in preeclampsia (PE) patients and controls. Comparison of sEng level between mild and sever PE patients with controls (A); early- and late-onset PE patients with controls (B) shows a significant increase in all PE groups in comparison with normal women. The 10^th^ and 90^th^ centiles are represented by lower and upper bars, and horizontal lines in the box show median. The circles represent outliers.

The mean serum level of sEng in women with mild PE was 24.08±3.05 ng/mL and in severe PE was 26.34±3.37 ng/mL as compared with 13.58±5.80 ng/mL in healthy controls. Preeclamptic women showed increased levels of sEng in both mild and severe groups compared to the control group (*P*<0.001). Also, a significant increase (*P*<0.001) was observed in the serum level of sEng in early-onset (26.10±2.68) and late-onset PE patients (24.62±3.47) in comparison with the control group (Fig. 2). In addition, there was no significant difference between mild and severe groups as well as the early- and late-onset groups (*P*=0.349 and *P*=1.0, respectively).

### Receiver-operating characteristic curve analysis

**Fig. 3:**
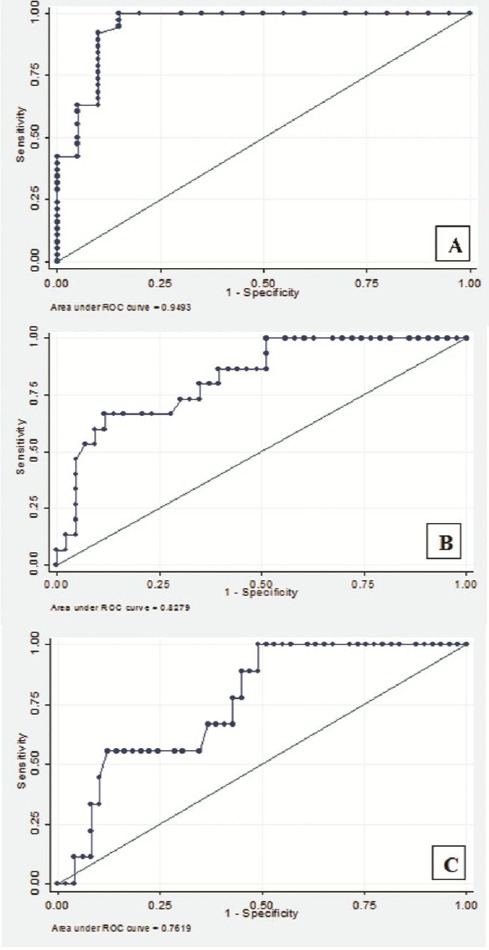
Performance of serum soluble endoglin (sEng) in diagnosis of preeclampsia (PE). Receiver-operating characteristic curve analysis of sEng levels in PE shows the ability of maternal serum sEng to differentiate: PE from normal pregnancies (A), severe PE patients (B), and early-onset PE patients (C).

The results of ROC curve analysis showed the highest diagnostic accuracy for sEng in diagnosis of preeclamptic patients from normal women with AUC of 0.94 (95% CI: 0.88-1.00), sensitivity and specificity of 92.1% and 90.0%, respectively. sEng had less accuracy in the detection of severe and early-onset PE patients (Fig. 3 and Table 3).

**Table 3:** The performance of serum endoglin in diagnosis of preeclampsia (PE)

Groups	cut off	Sn	Sp	LR+	LR-	PPV	NPV	Area (95% CI)
PE	≥20.4	92.1	90	**9.2**	0.08	94.6	85.7	0.94 (0.88 - 1.00)
Sever PE	≥24	80	65	2.2	0.30	44.4	90.3	0.82 (0.71 - 0.94)
Early-onset PE	≥24.4	66.6	63.2	1.8	0.52	25	91	0.76 (0.61 - 0.90)

Sn, sensitivity; Sp, specificity; LR, likelihood Ratio; PPV, positive predictive value; NPV, negative predictive value; CI, confidence interval

### Staining of endoglin in syncytiotrophoblast and cytotrophoblast cells of preeclampsia patients

**Fig. 4:**
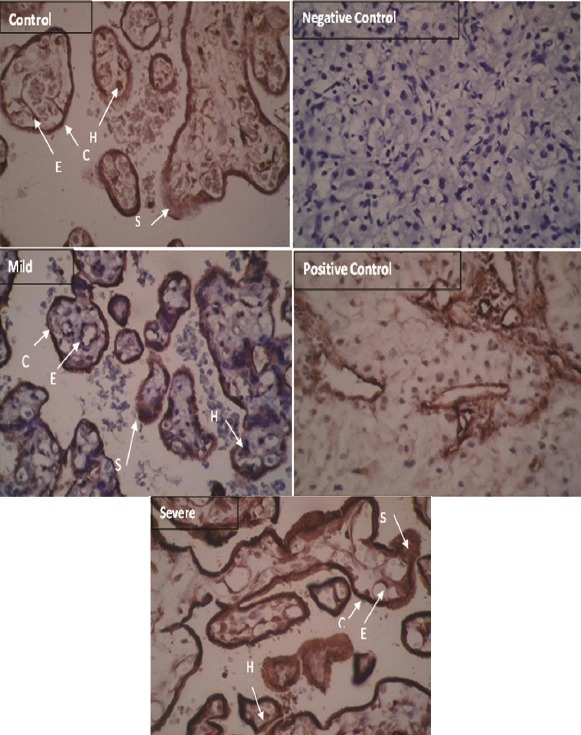
Immunohistochemistry of endoglin (Eng) in preeclampsia (PE) and control placenta. Eng is localized predominantly in syncytiotrophoblast and cytotrophoblast cells in PE patients. S, syncytiotrophoblast; C, cytotrophoblast; E, endothelial cells; H, Hofbauer cells (scale bar: 20 µm). Arrows show trophoblast cells.

Immunohistochemistry analysis showed severe intensity of staining which was observed pre-dominantly in syncytiotrophoblast and cytotrophoblast cells of severe PE patients and moderate intensity in placenta of mild PE patients. Eng was localized in placenta of controls with mild intensity of staining (Fig. 4 and Table 4).

**Table 4:** The membranous protein expression detected by immunohistochemistry in placenta tissue

Groups	Cytotrophoblast	Syncytiotrophoblast	Endothelial	Hofbauer
Control	+	+	-	-
Mild	++	++	+	+
Severe	+++	+++	++	+

(+), staining in more than 10% of cells with mild intensity; (++): moderate staining; (+++), severe staining. (-) is <10% intensity of staining

## DISCUSSION

Defect in angiogenesis, trophoblastic invasion, and uterine spiral artery remodeling are key events in the etiology of PE. Since TGF-β1 and its co-receptor, Eng, are involved in angiogenesis; therefore this hypothesis can be raised that alterations in expression of these genes have role in PE, and perhaps soluble form of Eng can be considered as a diagnostic biomarker for the identification of PE patients. According to our knowledge, this is the first study that investigates the TGF-β1 expression as well as soluble and membranous Eng expression in PE, particularly in placental tissue among Iranian population.

Our study did not show any significant changes in mRNA expression level of TGF-β1 in placental tissue of severe, mild, early- and late-onset PE, compared to healthy controls. However, there was only one study regarding TGF-β1 mRNA expression in placenta tissue of women affected with PE that reported an increased TGF-β1 mRNA expression in chorionic villous samples from pregnant women who suffered from PE later in the pregnancy[14].

Our data confirmed significant up-regulation of Eng mRNA in all patients, particularly with severe and early-onset of PE. These results are in accordance with the findings of other studies that reported up-regulation of Eng mRNA in PE[15-17]. Serum level of sEng was also evaluated, and a significant increase of sEng serum levels was found in all PE patients versus controls, while early-onset and severe PE patients had the highest sEng level. The results obtained for sEng level in PE are in line with those of numerous studies regarding this marker[1,9,18].

Angiogenesis defect in the early stages of pregnancy results in incomplete remodeling of spiral arterioles of uterus and abnormal placental vascular development as the main pathological finding in PE[8]. Eng is expressed in vascular endothelial cells, synctyiotrophoblasts, and invasive cytotrophoblasts and involved in placental implantation and spiral arterioles remodeling[11,19,20]. Eng regulates TGF-β1 signaling pathway by interacting with TGF-β1 receptor I and II, and also as a pro-angiogenic factor regulates nitric oxide-dependent vasodilation[21]. Overproduction of anti-angiogenic peptides like sEng is a response to placental ischemia, which is due to inadequate trophoblast invasion and contributes to the inhibition of angiogenesis[9,22]. Actually, sEng binds to TGF-β1 and prevents the attachment of TGF-β1 to its cell membrane receptors and inhibits the regulation of trophoblasts invasion[21]. The data of this study indicated that Eng mRNA and protein expression were increased in trophoblast cells (cytotrophoblasts and synctyiotrophoblasts) in PE patients. This phenomenon can be explained as a compensatory response of placenta to hypoxia. Also, results revealed that sEng was increased in the serum of PE women, which might be a potential diagnostic marker for PE in addition to blood pressure and proteinuria. AUC of 0.95 with more than 90% sensitivity and specificity refers to high accuracy of serum sEng in diagnosis of PE from normal pregnant women. Calculated positive and negative likelihood ratio and also positive and negative predictive value suggest sEng ability in this regard. Although sEng, based on our results, can be considered as a marker with high accuracy in identification of PE, this serum marker has lower accuracy for detecting severe and early-onset PE patients due to its lower sensitivity and specificity in the mentioned situations. De Vivo *et al*.[23] have studied on serum samples between 24 and 28 weeks of gestation and showed the usefulness of sEng in early prediction of PE with more than 80% sensitivity and specificity. Lim *et al*.[18] have reported AUC of 0.83 for sEng in predicting PE. Baumann *et al*.[24], reported AUC of 0.62 for sEng in predicting PE in first trimester serum in women with subsequent late-onset PE. Based on our ROC curve analysis, sEng has remarkable accuracy for diagnosis of PE from normal pregnant women compared with its ability for identification of severe and early-onset cases. Our main limitation was loss to follow-up patients from early pregnancy stages, before the onset of PE, and only patients diagnosed with PE were involved in our study as the case group. Therefore, evaluation of sEng, as a candidate marker, in a larger sample size and follow-up of patients from the earlier stages of pregnancy are suggested for longitudinal studies.

The present study showed the up-regulation of both membranous and soluble form of Eng in trophoblast cells of PE patients. Also, our data showed the usefulness of sEng, as a potential marker with high accuracy for diagnosis of PE and its severity. Prospective longitudinal studies in different populations are suggested to elucidate sEng as a diagnostic biomarker in PE patients.

## References

[1] Levine RJ, Lam C, Qian C, Yu KF, Maynard SE, Sachs BP, Sibai BM, Epstein FH, Romero R, Thadhani R, Karumanchi SA, CPEP Study Group (2006). Soluble endoglin and other circulating antiangiogenic factors in preeclampsia. The New England journal of medicine.

[2] Brownfoot F, Hannan N, Onda K, Tong S4, Kaitu’u-Lino T (2014). Soluble endoglin production is upregulated by oxysterols but not quenched by pravastatin in primary placental and endothelial cells. Placenta.

[3] Rana S, Powe CE, Salahuddin S, Verlohren S, Perschel FH, Levine RJ, Lim KH, Wenger JB, Thadhani R, Karumanchi SA (2012). Angiogenic factors and the risk of adverse outcomes in women with suspected preeclampsia. Circulation.

[4] Wu P, van den Berg C, Alfirevic Z, O’Brien S, Röthlisberger M, Baker PN, Kenny LC, Kublickiene K, Duvekot JJ (2015). Early pregnancy biomarkers in pre-eclampsia:a systematic review and meta-analysis. International journal of molecular sciences.

[5] Kaitu’u-Lino T, Hastie R, Cannon P, Nguyen H, Lee S, Hannan NJ, Tong S (2015). Transcription factors E2F1 and E2F3 are Kaitu’u expressed in placenta but do not regulate MMP14. Placenta.

[6] Kar M (2014). Role of biomarkers in early detection of preeclampsia. Journal of clinical and diagnostic research.

[7] Wang Y, Wang Q, Guo C, Wang S, Wang X, An L, Cao X, Qiu Y, Wang G, Li H, Ma X (2014). Association between CRP gene polymorphisms and the risk of preeclampsia in Han Chinese women. Genetic testing and molecular biomarkers.

[8] Wolf M, Hubel CA, Lam C, Sampson M, Ecker JL, Ness RB, Rajakumar A, Daftary A, Shakir AS, Seely EW, Roberts JM, Sukhatme VP, Karumanchi SA, Thadhani R (2004). Preeclampsia and future cardiovascular disease:potential role of altered angiogenesis and insulin resistance. The journal of clinical endocrinology and metabolism.

[9] Aggarwal P, Chandel N, Jain V, Jha V (2010). The relationship between circulating endothelin-1, soluble fms-like tyrosine kinase-1 and soluble endoglin in preeclampsia. Journal of human hypertension.

[10] Perucci LO, Gomes KB, Freitas LG, Godoi LC, Alpoim PN, Pinheiro MB, Miranda AS, Teixeira AL, LM Dusse, Sousa LP (2014). Soluble endoglin, transforming growth factor-Beta 1 and soluble tumor necrosis factor alpha receptors in different clinical manifestations of preeclampsia. PloS one.

[11] Bell MJ, Roberts JM, Founds SA, Jeyabalan A, Terhorst L, Conley YP (2013). Variation in endoglin pathway genes is associated with preeclampsia:a case–control candidate gene association study. BMC pregnancy and childbirth.

[12] (2002). American College of Obstetricians and Gynecologsts:ACOG practice bulletin 33:diagnosis and management of preeclampsia and eclampsia. Obstetrics and Gynecology.

[13] Von Dadelszen P, Magee LA, Roberts JM (2003). Subclassification of preeclampsia. Hypertension in pregnancy.

[14] Farina A, Sekizawa A, De Sanctis P, Purwosunu Y, Okai T, Cha DH, Kang JH, Vicenzi C, Tempesta A, Wibowo N, Valvassori L, Rizzo N (2008). Gene expression in chorionic villous samples at 11 weeks’gestation from women destined to develop preeclampsia. Prenatal diagnosis.

[15] Purwosunu Y, Sekizawa A, Yoshimura S, Farina A, Wibowo N, Nakamura M, Shimizu H, Okai T (2009). Expression of angiogenesis-related genes in the cellular component of the blood of preeclamptic women. Reproductive sciences.

[16] Sekizawa A, Purwosunu Y, Farina A, Shimizu H, Nakamura M, Wibowo N, Rizzo N, Okai T (2010). Prediction of pre-eclampsia by an analysis of placenta-derived cellular mRNA in the blood of pregnant women at 15–20 weeks of gestation. BJOG.

[17] Sitras V, Paulssen RH, Grønaas H, Leirvik J, Hanssen TA, Vårtun A, Acharya G (2009). Differential placental gene expression in severe preeclampsia. Placenta.

[18] Lim JH, Kim SY, Park SY, Yang JH, Kim MY, Ryu HM (2008). Effective prediction of preeclampsia by a combined ratio of angiogenesis-related factors. Obstetrics and gynecology.

[19] Caniggia I, Taylor CV, Ritchie JW, Lye SJ, Letarte M (1997). Endoglin regulates trophoblast differentiation along the invasive pathway in human placental villous explants. Endocrinology.

[20] Toporsian M, Gros R, Kabir MG, Vera S, Govindaraju K, Eidelman DH, Husain M, Letarte M (2005). A role for endoglin in coupling eNOS activity and regulating vascular tone revealed in hereditary hemorrhagic telangiectasia. Circulation research.

[21] Lim JH, Kim SY, Park SY, Lee HM, Yang JH, Kim MY, Chung JH, Lee SW, Ryu HM (2009). Soluble endoglin and transforming growth factorβ1 in women who subsequently developed preeclampsia. Prenatal diagnosis.

[22] Hawinkels LJ, Kuiper P, Wiercinska E, Verspaget HW, Liu Z, Pardali E, Sier CF, ten Dijke P (2010). Matrix metalloproteinase-14 (MT1-MMP)–mediated endoglin shedding inhibits tumor angiogenesis. Cancer research.

[23] De Vivo A, Baviera G, Giordano D, Todarello G, Corrado F, D’anna R (2008). Endoglin, PlGF and sFlt-1 as markers for predicting pre-eclampsia. Acta obstetricia et gynecologica scandinavica.

[24] Baumann MU, Bersinger NA, Mohaupt MG, Raio L, Gerber S, Surbek DV (2008). Firs-trimester serum levels of soluble endoglin and soluble fms-like tyrosine kinase-1 ats first-trimester markers for late-onset preeclampsia. American journal of obstetrics and gynecology.

